# Auditing Road-Segment Speed Forecasting Under Sparse Mobile Probe Sensing: A Mask-Consistent Support-Chain Analysis

**DOI:** 10.3390/s26134320

**Published:** 2026-07-07

**Authors:** Dingxin Wu, Zheng Xu, Zhiyuan Wang, Kai Huang, Hong Ki An, Dewen Kong

**Affiliations:** 1 Faculty of Transportation Engineering, Huai’an University, Huai’An 223001, China; 2 Faculty of Computer & Software Engineering, Huai’an University, Huai’An 223001, China; 212409611261@hau.edu.cn; 3 School of Instrument Science and Engineering, State Key Laboratory of Comprehensive PNT Network and Equipment Technology, Southeast University, Nanjing 211189, China; kaihuang@seu.edu.cn; 4 School of Civil Engineering, Universiti Sains Malaysia, Engineering Campus, Nibong Tebal 14300, Pulau Pinang, Malaysia; anhongki77@usm.my; 5 Beijing Key Laboratory of Traffic Engineering, Beijing University of Technology, Beijing 100124, China; kongdw@bjut.edu.cn

**Keywords:** mobile probe sensing, sparse observations, road-segment speed forecasting, mask-consistent evaluation, evaluation support, common support, congestion diagnosis, intelligent transportation systems

## Abstract

**Highlights:**

**What are the main findings?**

- A support-chain audit traces how sparse probe sensing contracts evaluable speed-forecasting targets from the full grid to observed, model-valid, and common-support subsets.
- Under the relaxed Target-observed support, the adaptive graph convolutional recurrent network (AGCRN) shows the smallest mean absolute error (MAE) and strongest congestion F1 score (harmonic mean of precision and recall) among the evaluated full-coverage models, whereas the historical mean (HIST_MEAN) baseline shows the smallest root mean squared error (RMSE); these complementary outcomes should be interpreted as metric-dependent and support-conditioned evidence rather than as a single universally superior ranking.

**What are the implications of the main findings?**

- The Transformer-LSTM (TT-LSTM) probe features provide only small global accuracy gains; congestion recall remains low for all full-coverage deep models under sparse sensing.
- Reporting evaluation support is critical for fair benchmarking when forecasting depends on sparse and uneven mobile sensing data.

**Abstract:**

Ride-hailing global positioning system (GPS) mobile probe data provide flexible urban traffic observations, but their sparse and uneven coverage makes model evaluation difficult because observed targets, valid predictions, and historical input support do not always coincide. This study audits ultra-short-term road-segment speed forecasting under sparse mobile sensing using a mask-consistent support-chain framework. A three-day GPS dataset is aggregated into 5 min speed observations over 1970 road segments and used as a controlled sparse-sensing case study rather than a general-purpose long-term forecasting benchmark. The evaluation protocol distinguishes the full test grid, the set of directly observed target speeds, model-valid prediction support, strict complete-history support, and common-support subsets for coverage-limited baselines. The directly observed target set is used as the primary relaxed support because it retains all verifiable ground-truth targets, while strict and common-support subsets are reported as sensitivity checks. Under this support-conditioned evaluation, the adaptive graph convolutional recurrent network (AGCRN) is associated with lower mean absolute error (MAE) among full-coverage models, the historical mean (HIST_MEAN) baseline is associated with lower root mean squared error (RMSE), and congestion recall remains below 0.24 for all full-coverage deep models. These complementary results indicate conditional and metric-dependent strengths rather than universal model superiority. Because the dataset covers only three consecutive days, weekday/weekend variation, incident-specific fluctuations, seasonal effects, and spatial transferability cannot be fully examined and are treated as limitations. Overall, the findings show that evaluation support should be reported as a first-order experimental factor alongside model accuracy under sparse mobile probe sensing.

## 1. Introduction

Road-segment speed forecasting is a fundamental task in intelligent transportation systems because it supports traffic monitoring, travel-time estimation, congestion diagnosis, route guidance, and proactive traffic management [1,2]. Recent studies on heterogeneous sensing and multi-sensor speed or travel-time estimation further show that urban traffic states can be inferred from diverse sensing sources [3,4]. With the increasing availability of connected vehicles, ride-hailing fleets, taxis, and other global positioning system (GPS)-equipped vehicles, mobile probe sensing has become an important complement to fixed roadside infrastructure [5]. Compared with loop detectors, cameras, and other fixed sensors, mobile probes provide flexible and low-cost observations over large urban road networks. However, this sensing mechanism is opportunistic rather than continuous: a road segment is observed only when one or more probe vehicles pass through it during a given time interval. Consequently, mobile probe sensing naturally produces sparse, uneven, and spatially biased road segment–time observations.

This sparse sensing mechanism creates a central difficulty for road-segment speed forecasting. In conventional fixed-sensor benchmarks, each sensor usually provides a relatively regular time series, and the main task is to model spatial and temporal dependencies among observed sensors. In mobile-probe-based road networks, each road segment acts as a virtual sensor whose observation frequency depends on probe penetration, travel demand, road class, time of day, and spatial accessibility. Therefore, the resulting speed matrix contains many missing entries, and a large proportion of road segment–time positions have no directly observed ground-truth speed. Studies on sparse mobile crowdsensing and sparse movement-speed forecasting show that mobile probe sensing creates structurally incomplete observations [5,6]. Related work on sparse segment-network prediction and sparse probe speed prediction further shows that such sparsity affects road-segment forecasting and evaluation support [7,8,9]. This raises the following specific research question: under sparse mobile probe sensing, how should road-segment speed forecasting models be evaluated when observed targets, valid predictions, and historical input support do not coincide?

Traffic forecasting methods have developed rapidly in recent years. Graph neural networks and other deep spatiotemporal models have become representative tools because transportation networks naturally exhibit graph-structured spatial dependencies, while traffic states evolve dynamically over time. Recent surveys and progress reviews have summarized graph construction strategies, spatial–temporal modeling architectures, datasets, and open-source implementations for traffic forecasting [10,11,12]. Representative architectures—including diffusion convolutional recurrent neural networks (DCRNN), spatiotemporal graph convolutional networks (STGCN), Graph WaveNet (GWNet), and adaptive graph convolutional recurrent networks (AGCRN)—have demonstrated strong performance across traffic forecasting benchmarks [13,14,15,16]. These developments indicate that architecture-oriented traffic forecasting research has become highly mature.

Nevertheless, a model-centered perspective is not sufficient under sparse mobile probe sensing. When ground-truth observations are incomplete, reported errors may depend not only on forecasting ability but also on the evaluation support on which errors are computed. If different models or baselines are evaluated on different road segment–time positions, their mean absolute error (MAE), root mean squared error (RMSE), or congestion metrics may no longer be directly comparable. This issue is especially important when full-coverage models are compared with coverage-limited baselines such as persistence or DailyProfile predictors. Such baselines are usually valid only at positions with recent or historical observations, and these valid subsets may be biased toward observation-dense and temporally regular traffic states. Without explicitly auditing the evaluated targets, a comparison may conflate forecasting performance with coverage bias.

In the traffic missing-data literature, the dominant focus is on reconstructing or completing unobserved values. Recent survey and benchmarking studies have systematized this area by evaluating spatial–temporal traffic-data imputation models under diverse missing patterns and missing rates [17,18]. Tensor-completion and neural imputation methods further provide concrete mechanisms for recovering missing traffic states under structured missingness [19,20,21]. These studies are valuable when the objective is to reconstruct a more complete traffic state matrix or improve downstream prediction under missing inputs. However, forecasting evaluation under naturally sparse mobile probe observations requires an orthogonal audit of which naturally observed targets can be directly assessed without imputation. If an unobserved target is filled and then used as evaluation truth, the evaluation may obscure whether the target was directly observed by the sensing system. Therefore, beyond reconstructing missing traffic states, it is necessary to audit which naturally observed targets can be directly evaluated under mask-consistent conditions.

Benchmarking and reproducibility have also received increasing attention in time-series and spatial–temporal forecasting. Modular benchmark frameworks for spatial–temporal networks and broader Time-Series Forecasting Benchmarks have promoted fairer comparisons by standardizing datasets, model components, training procedures, and evaluation pipelines [22,23]. These efforts address important issues such as inconsistent implementations and limited evaluation protocols. However, sparse mobile probe sensing introduces an additional support-level problem: even when the experimental pipeline is standardized, models and baselines can only be fairly compared when their errors are computed over the same set of observed road segment–time targets.

This study audits road-segment speed forecasting under sparse mobile probe sensing using a mask-consistent support-chain analysis rather than proposing another forecasting architecture. The empirical analysis uses a three-day ride-hailing GPS dataset aggregated into 5 min road-segment speeds over an urban road network, with each road segment treated as a virtual sensor. The framework separates the full test grid, Target-observed support, strict Evaluation-eligible support, own-valid baseline support, and DailyProfile-common support so that model comparison, baseline interpretation, congestion diagnosis, and ablation analysis are all tied to explicit evaluation boundaries. The relaxed Target-observed support is used as the primary evaluation set because it retains all directly observed ground-truth targets, while the strict complete-history subset and baseline-inclusive common support are reported as sensitivity checks. Within this design, representative graph-based deep learning models, classical and tabular baselines, and a lightweight Transformer-LSTM (TT-LSTM) analytical probe are evaluated under consistent target masks; TT-LSTM is used specifically to diagnose the practical value of hand-crafted sparse-input features rather than to serve as a new state-of-the-art model.

The contributions and findings are therefore summarized as follows. First, this study introduces a mask-consistent support-chain audit that decouples target observability, input eligibility, strict evaluation eligibility, own-valid baseline support, and common-support comparison under sparse mobile probe sensing. Second, it shows that full-coverage model rankings and coverage-limited baseline results must be interpreted together with their evaluation support, because mixing different supports can create coverage bias. Third, the TT-LSTM probe indicates that simple sparse-input feature engineering provides only limited global benefit, while congestion detection remains difficult for full-coverage deep models. Overall, the case study supports the central methodological conclusion that evaluation support, prediction coverage, and support-induced bias should be reported transparently alongside model accuracy.

The remainder of this paper is organized as follows. Section 2 reviews related work on mobile probe sensing, graph-based traffic forecasting, missing traffic data, and benchmarking under incomplete observations. Section 3 describes the data, topology-proximity graph, regional organization, input feature construction, TT-LSTM probe model, missing-history handling, and evaluation support-chain design. Section 4 presents the support-chain audit, relaxed primary evaluation, coverage-limited baseline analysis, feature ablation, congestion diagnosis, boundary analysis, and strict-subset sensitivity. Section 5 discusses the implications and limitations of the findings. Section 6 concludes the paper.

## 2. Related Work

### 2.1. Mobile Probe Sensing and Sparse Road-Segment Observations

Urban traffic states can be observed through different sensing mechanisms. Fixed sensors, such as loop detectors, cameras, radar detectors, and point speed sensors, provide relatively regular temporal measurements at installed locations, but their spatial coverage is limited by deployment cost and infrastructure availability. In contrast, mobile probe sensing uses GPS-equipped vehicles, taxis, ride-hailing fleets, or other connected vehicles as moving observation units. This sensing paradigm provides flexible and low-cost spatial coverage over large urban road networks and has been increasingly used for travel-time prediction, traffic state estimation, multi-sensor speed estimation, sparse movement-speed forecasting, and sparse probe speed prediction [3,4,5,6,7,8,9].

Unlike fixed sensors that continuously monitor the same location, mobile probe sensing is opportunistic: a road segment is observed only when a probe vehicle traverses it, creating a distributed but intermittent sensing system in which each segment serves as a virtual sensor. This virtual-sensor perspective is useful for road-segment speed forecasting because it allows GPS trajectories to be organized into a segment–time speed matrix. However, the resulting matrix is naturally sparse, uneven, and spatially biased.

Several factors produce this sparsity: probe penetration varies across regions and time periods; travel demand concentrates on certain corridors; road class affects the likelihood of ride-hailing or taxi traversal; and night-time, low-demand, or peripheral segments often have very few observations. As a result, the absence of an observed speed is not merely random measurement noise. It reflects the sensing mechanism itself. Studies on sparse mobile crowdsensing, sparse movement-speed forecasting, sparse segment-network prediction, and sparse probe speed prediction have shown that this type of sparsity is a defining property of mobile-probe-based traffic data rather than a secondary preprocessing problem [5,6,7,8,9].

This observation has direct implications for evaluation. In fixed-sensor datasets, most sensor–time positions are usually assumed to be observed or recoverable. In mobile-probe-based road networks, however, a large fraction of road segment–time positions may have no directly observed ground-truth speed. This opportunistic observation mechanism implies that not all road segment–time positions are equally observable or equally evaluable, which motivates an explicit audit of evaluation support, see Table 1.

### 2.2. Road-Segment Traffic Forecasting and Spatiotemporal Graph Models

Traffic forecasting has evolved from classical statistical models and sequence-based neural networks to graph-based and attention-based spatiotemporal models. Early deep learning approaches mainly focused on temporal dependency modeling through recurrent or convolutional sequence architectures. As transportation networks are naturally graph-structured, later research increasingly incorporated road topology, spatial proximity, and learned spatial dependencies into forecasting models. Recent surveys have summarized this development and shown that graph neural networks have become a representative class of models for traffic forecasting tasks [10,11,12,24,25].

Graph-based message passing provides a natural way to model spatial interactions among traffic sensors or road segments. Diffusion-based recurrent models represent traffic propagation as a diffusion process on a directed graph, while spatiotemporal graph convolutional models combine temporal convolution with graph convolution to capture joint spatial and temporal dependencies [13,14]. Graph WaveNet (GWNet) further relaxes reliance on predefined topology by learning adaptive dependencies, and the adaptive graph convolutional recurrent network (AGCRN) introduces node-adaptive parameter learning to capture heterogeneous spatial patterns [15,16]. These models show the value of graph-based spatial representations for traffic forecasting.

Recent developments have further expanded this architecture-oriented research line. A diverse array of architectures—including dynamic and synchronous spatiotemporal graphs, graph ordinary differential equations, identity-based baselines, and Transformer-based mechanisms—has been proposed to capture more complex traffic dynamics [24,25,26,27,28,29,30,31,32]. For example, the decoupled dynamic spatial–temporal graph neural network (D2STGNN) explicitly decouples traffic signals into different dynamic components [27], while spatial–temporal identity (STID) shows that simple spatial–temporal identity representations can also form strong baselines under certain benchmark settings [30]. Transformer-based architectures such as the propagation delay-aware dynamic long-range Transformer (PDFormer) [31] and the spatiotemporal adaptive embedding Transformer (STAEformer) [32] further push traffic forecasting toward long-range dependency modeling and adaptive embedding design. These advances indicate that traffic forecasting research has become highly mature at the model-design level.

However, most of these studies primarily focus on improving spatiotemporal representation learning, graph construction, adaptive dependency modeling, or temporal attention mechanisms. Evaluation is typically conducted on predefined benchmark splits, often based on fixed sensors or relatively regular observation grids. Under sparse mobile probe sensing, therefore, comparing these strong forecasting models fairly still requires explicit support-level auditing, because reported errors may conflate model capacity with differences in the evaluation targets themselves.

### 2.3. Missing Traffic Data: Imputation, Completion, and Sparse-Input Prediction

Missing traffic data have long been studied in intelligent transportation systems. Missingness can arise from sensor malfunction, communication failures, low sampling frequency, insufficient probe penetration, or incomplete spatial coverage. A large body of research has therefore focused on reconstructing missing traffic states through imputation, completion, or recovery methods. Recent surveys have systematized this field by evaluating spatiotemporal traffic-data imputation methods under different missing patterns and missing rates [17,18].

Existing missing-data studies can be broadly grouped into several directions. Matrix and tensor completion methods exploit low-rank structures and temporal regularities in traffic matrices. Graph-based imputation methods use road network topology or learned spatial relations to propagate information from observed positions to missing ones. Deep learning methods combine recurrent networks, graph neural networks, attention mechanisms, and generative models to infer unobserved traffic states. Other studies incorporate uncertainty-aware mechanisms, missing-data completion models, imputation systems, and missing-data-aware prediction architectures to improve robustness when historical inputs are incomplete [19,20,21,33,34,35]. For instance, coupling long short-term memory (LSTM) with graph Laplacian regularized matrix factorization combines temporal prediction and spatial smoothness for imputation [33], truncated tensor Schatten p-norm models address complex missing patterns through tensor completion [34], and mixture-of-experts architectures have been introduced to handle heterogeneous missingness during reconstruction [35].

These methods are valuable when the goal is to reconstruct a complete traffic state matrix or improve prediction under missing inputs. For operational estimation or visualization, imputing missing speed values may be useful and even necessary. For forecasting evaluation, however, using imputed values as ground truth may partly measure agreement with the imputation procedure rather than accuracy against directly sensed traffic states. This distinction is especially important for mobile probe data, where the absence of a speed observation means that the sensing system did not directly observe the road segment during that time interval.

Therefore, the present study does not treat missing target values as quantities to be filled for evaluation. Instead, it treats target observability as an explicit evaluation condition. Imputation-oriented studies ask how missing traffic states can be reconstructed; this study asks which naturally observed targets can be directly evaluated. This difference motivates the mask-consistent support-chain perspective adopted in this paper.

### 2.4. Benchmarking, Evaluation Support, and Mask-Consistent Comparison

Benchmarking and reproducibility have become increasingly important in time-series and spatiotemporal forecasting. Recent benchmark frameworks aim to standardize datasets, model implementations, training procedures, hyperparameter settings, and evaluation metrics, thereby improving fairness and reproducibility across forecasting studies. For example, Spatiotemporal Gym (STGym) provides a modular benchmark for spatiotemporal networks with a traffic forecasting case study, while Time Series Forecasting Benchmark (TFB) emphasizes comprehensive and fair benchmarking of time-series forecasting methods across datasets and model families [22,23]. Imputation benchmarks have also begun to evaluate missing-data methods under controlled missing patterns and missing rates [17,36]. For example, Time Series Imputation Benchmark (TSI-Bench) provides a systematic evaluation of time-series imputation methods and highlights the importance of standardized evaluation protocols [36].

These efforts improve what can be called pipeline-level fairness. They help reduce discrepancies caused by inconsistent data splits, implementation details, optimization settings, and metric definitions. However, sparse mobile probe sensing introduces an additional support-level problem. Even if models are trained and evaluated under the same software framework, they may still be evaluated on different road segment–time positions if their prediction coverage differs or if some baselines are only valid under certain historical-observation conditions.

This issue is especially important when comparing full-coverage models with coverage-limited baselines. A deep learning model may generate predictions for all observed targets after missing input positions are handled through a uniform masking or filling protocol, while a persistence baseline may only be valid when the immediately previous time step is observed. A DailyProfile baseline may only be valid when the same road segment and time slot have sufficient historical observations. These own-valid subsets are not random samples of the test grid; they often concentrate on observation-dense, temporally regular, or frequently traversed road segments. As a result, their errors and congestion metrics may not be directly comparable with those of full-coverage models evaluated on all observed targets.

Existing benchmarking efforts standardize the experimental machinery, but they do not necessarily guarantee that the road segment–time positions over which errors are computed are identical for all compared methods. Under sparse mobile probe sensing, this support-level comparability becomes a prerequisite for interpreting model rankings, baseline competitiveness, and congestion metrics. This motivates a mask-consistent evaluation design that explicitly separates target observability, input eligibility, prediction coverage, and baseline-common support.

### 2.5. Summary of Research Gaps

The literature reviewed above reveals three gaps that motivate this study.

**Gap 1: Sparse sensing is often treated as missing-data reconstruction rather than an evaluation-support problem.** Mobile probe observations are naturally sparse because vehicles act as opportunistic moving sensors. Existing missing-data studies mainly focus on imputing or completing unobserved traffic states, but fewer studies treat the set of naturally observed targets as an experimental object that must be explicitly reported and audited.

**Gap 2: Model comparisons often assume comparable evaluation targets.** Spatiotemporal graph models and Transformer-based predictors have achieved strong performance in traffic forecasting, but their evaluations usually rely on predefined benchmark splits or relatively regular sensing grids. Under sparse mobile probe sensing, whether different models are evaluated on the same road segment–time targets is not a trivial implementation detail but a core condition for fair comparison.

**Gap 3: Coverage-limited baselines are rarely separated clearly from full-coverage models.** Persistence, DailyProfile, and other history-dependent baselines may only produce predictions on subsets with sufficient historical observations. These subsets can be biased toward dense and regular traffic patterns. If their own-valid results are mixed with full-coverage model results, baseline competitiveness and congestion metrics may be misinterpreted.

To address these gaps, this paper constructs a mask-consistent support-chain audit and applies it to road-segment speed forecasting under sparse mobile probe observations. The audit is then used to diagnose full-coverage model comparison, coverage-limited baselines, congestion detection, and sparse-input feature ablations.

Taken together, the literature reviewed above shows that traffic prediction is an established intelligent transportation systems (ITS) task, graph-based forecasting provides mature spatial–temporal modeling tools, and sparse mobile sensing creates an evaluation-support problem that cannot be resolved by model comparison alone, see Table 2.

## 3. Methods

**Figure 1:**
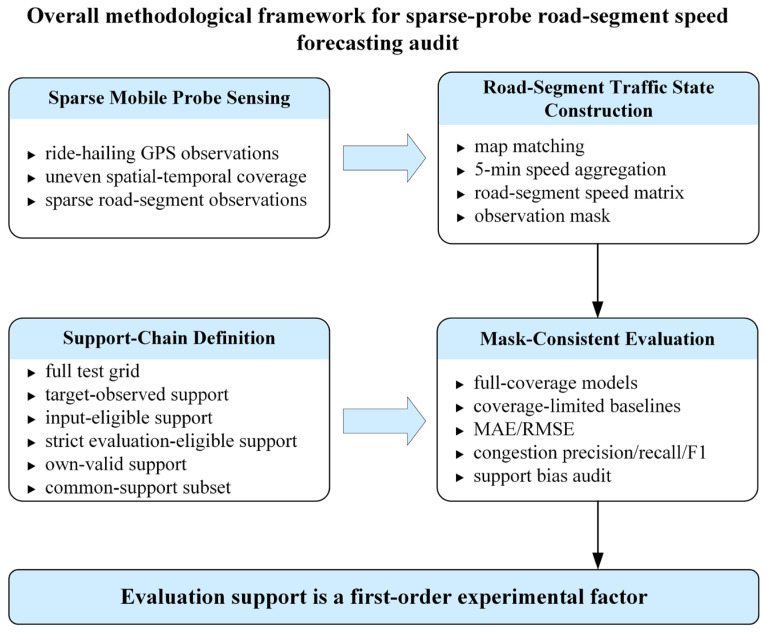
Overall methodological workflow.

Figure 1 summarizes the full analysis pipeline from raw ride-hailing GPS observations to mask-consistent evaluation. The workflow emphasizes that data aggregation, observation-mask construction, model prediction, and support-specific evaluation are treated as linked stages rather than independent preprocessing and modeling steps.

**Figure 2:**
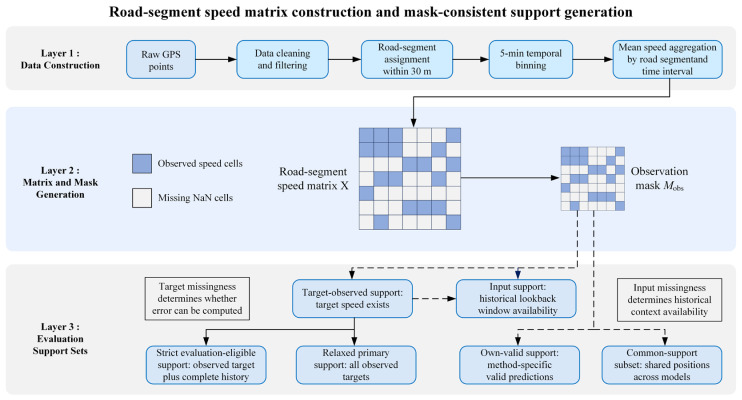
Road-segment speed matrix construction and mask generation. Solid arrows indicate the main data-processing flow, whereas dashed arrows indicate auxiliary or optional information flow.

Figure 2 illustrates how raw GPS trajectories are matched to road segments and aggregated into 5 min segment-level speed observations. Missing segment-time cells define the observation mask, which further determines Target-observed support, input support, and strict evaluation eligibility.

### 3.1. Data Preparation and Road-Segment Speed Matrix

This study uses three consecutive days of ride-hailing GPS trajectory observations to construct road-segment-level speed observations over an urban arterial and sub-arterial road network. The three-day horizon is used to audit evaluation-support behavior under a controlled sparse-sensing setting rather than to estimate weekly periodicity or long-term traffic seasonality. The raw GPS observations are first projected into Universal Transverse Mercator (UTM) Zone 50 N to enable metric-distance calculations, and road-segment association is performed using deterministic nearest-segment spatial assignment. Specifically, each GPS point is assigned to the nearest candidate road segment when the perpendicular distance is within 30 m. Points outside this threshold are discarded to reduce spatial assignment noise. This deterministic assignment is used to construct segment-level observations for the support-chain audit, rather than to optimize map-matching performance.

The trajectory observations are aggregated into 5 min time intervals. For each road segment and each time interval, observed vehicle speeds assigned to that segment are averaged to obtain a segment-level speed value. The resulting observation period contains T=864 time steps, corresponding to three days of 5 min intervals. The final experimental road-segment set contains N=1970 road segments. All speed values are converted to m/s before model training and evaluation.

The three-day observation period is split into two training days and one test day. In the implementation, zero-based time indices 0–575 are used for training, and indices 576–863 are used as the test period. Equivalently, under the one-based notation used in the equations below, the first 576 time steps are used for training, and the final 288 time steps are used for testing. Therefore, the test period contains $|T_{test}|=288$ five-minute intervals.

The resulting road-segment speed matrix is denoted as

(1) $$X\in {\mathbb{R}}^{T\times N},$$

with elements

(2) $$X={\left\{x_{t,i}\right\}}_{t=1,i=1}^{T,N},$$

where $x_{t,i}$ denotes the observed speed of road segment i at time step t.

Because mobile probe sensing is sparse and uneven, many entries in X are unavailable. Therefore, we define a binary validity mask

(3) $$m_{t,i}=\left\{\begin{array}{cc} 1, & \mathrm{if}\;x_{t,i}\;\mathrm{is}\;\mathrm{finite}\;\mathrm{and}\;\mathrm{observed},\\ 0, & \mathrm{otherwise}. \end{array}\right.$$

The corresponding mask matrix is denoted as $M\in {\left\{0,1\right\}}^{T\times N}$. We use $M_{t-L:t-1}$ to denote the restriction of the full validity mask to the historical input window. In this study, missing entries in the target matrix are not treated as ground-truth labels. They are excluded from loss and metric computation through the mask-consistent evaluation protocol described in Section 3.8.

### 3.2. Forecasting Task and Mask Notation

The forecasting task is formulated as single-step road-segment speed prediction. Given a historical lookback window of length L=12, corresponding to 60 min of historical observations, the model predicts the speed vector at the next 5 min time step.

For a prediction time t, the target is

(4) $$x_{t}={\left[x_{t,1},x_{t,2},\ldots ,x_{t,N}\right]}^{⊤}$$

The historical input window is

(5) $$X_{t-L:t-1}=\left\{x_{t-L},x_{t-L+1},\ldots ,x_{t-1}\right\}.$$

For graph-based forecasting models, the prediction can be written as

(6) $${\hat{x}}_{t}=f_{\theta }\left(X_{t-L:t-1},A,M_{t-L:t-1}\right),$$

where A denotes the topology-proximity adjacency matrix or its learned variant, depending on the model. Different graph-based models may use A differently, but the forecasting target and evaluation mask remain the same.

For the TT-LSTM probe model, the input is expressed using a feature tensor

(7) $${\hat{x}}_{t}=f_{\theta }\left(Z_{t-L:t-1}\right),$$

where $Z_{t-L:t-1}$ contains raw speed values, topology-derived k-hop statistics, input validity masks, and causal multi-scale temporal features. The construction of Z is detailed in Section 3.4.

During training and evaluation, unobserved targets are not used as supervised labels. For a support set S, the masked loss is computed only over valid target positions:

(8) $$L_{S}=\frac{1}{|S|}\underset{\left(t,i\right)\in S}{\sum }l({\hat{x}}_{t,i},x_{t,i}),$$

where $l\left(\cdot \right)$ denotes the pointwise prediction loss. In all cases, the target position $\left(t,i\right)$ must have an observed ground-truth speed value.

Table 3 reports the training and testing settings used in the final forecasting experiments. These settings are included to make the comparison reproducible and to clarify the temporal split, look-back window, forecasting horizon, evaluation support, optimizer, learning rate, batch size, and hardware environment.

### 3.3. Topology-Proximity Graph and Regional Organization

#### 3.3.1. Topology-Proximity Graph

A topology-proximity graph is constructed to represent structural relations among road segments. The graph is denoted as

(9) $$G=\left(V,E\right),$$

where each node in V represents a candidate road segment and each edge in E represents a structural or spatial relation between two road segments. Two types of relations are considered. First, endpoint-sharing topology is used to connect road segments that are adjacent in the road network. Second, spatial proximity is used to connect segments whose endpoints or geometries are within 30 m. This design captures both explicit network connectivity and local geometric proximity.

The constructed graph contains 8510 candidate road-segment nodes and 66,798 edges, with an average degree of approximately 7.8. The corresponding adjacency matrix is denoted as A. In the experiments, the final forecasting and evaluation set contains 1970 road segments selected from this candidate road network after regional organization and data availability filtering.

The topology-proximity graph serves three purposes in this study. First, it provides structural input for graph-based forecasting baselines. Second, it supports the construction of spatially coherent regional modeling units. Third, it provides the source graph for computing k-hop diagnostic local-context statistics used by the TT-LSTM probe model. The equal-weight k-hop features derived from this graph are used solely for diagnostic purposes and do not imply that such spatial averaging is inherently beneficial under sparse observations.

#### 3.3.2. Structure-Aware Regional Organization

The large sparse virtual-sensor network is organized into spatially coherent modeling units through a structure-aware regional organization procedure. The purpose of this step is to improve computational manageability and to group road segments with related spatial and temporal characteristics. It is used as an engineering organization strategy rather than as a claim of model-level novelty.

Temporal speed profiles are used to extract representative traffic patterns. Non-negative matrix factorization (NMF) is applied with temporal components, non-negative double singular value decomposition with averaging (NNDSVDa) initialization, and random seed 42. Missing values are temporarily filled solely for the NMF embedding step; these filled values are never used as forecasting targets or evaluation ground truth.

Before regional organization, the three-day average speed field exhibits strong positive spatial autocorrelation under the topology-proximity graph, with Moran’s I=0.7253. This supports the use of spatially coherent regional modeling units. The temporal-pattern representation is combined with spatial contiguity constraints to form 22 spatially contiguous regions, a number determined empirically to balance region size and within-region homogeneity. The resulting regions contain approximately 33 to 274 road segments each. These regions are used for region-wise model training and inference, especially for the TT-LSTM probe model and its no-partition control. This organization allows the forecasting task to be conducted on manageable subgraphs while preserving spatial coherence.

### 3.4. Input Feature Construction

The TT-LSTM probe model uses explicitly constructed input features to diagnose whether simple sparse-input feature engineering is useful under mobile probe sensing. Three feature configurations are considered: C = 4, C = 5, and C = 7.

For the C = 4 configuration, four diagnostic local-context features are constructed for each historical time step τ and road segment i: raw observed speed $x_{\tau ,i}$; 1-hop neighborhood mean; 2-hop neighborhood mean; residual between raw speed and 1-hop mean.

Let $N^{\left(k\right)}\left(i\right)$ denote the k-hop neighborhood of road segment i derived from the topology-proximity graph. The k-hop neighborhood mean is computed as

(10) $$\mu_{\tau ,i}^{\left(k\right)}=nanmean\left\{x_{\tau ,j}:j\in N^{\left(k\right)}\left(i\right)\right\},k\in \left\{1,2\right\}.$$

The residual feature is defined as

(11) $$r_{\tau ,i}=x_{\tau ,i}-\mu_{\tau ,i}^{\left(1\right)}.$$

If all observations in a k-hop neighborhood are missing at time τ, the corresponding neighborhood mean remains NaN and is subsequently handled by the missing-input strategy described in Section 3.6. Equal-weight k-hop statistics are included as diagnostic local-context features to test whether simple local spatial averaging is effective under sparse mobile probe observations. These k-hop statistics serve as a diagnostic local-context baseline: both positive and negative ablation outcomes are informative for assessing whether equal-weight spatial averaging is suitable under sparse mobile probe observations.

The C = 5 configuration adds an input validity mask channel:

(12) $$v_{\tau ,i}=\left\{\begin{array}{cc} 1, & \mathrm{if}\;x_{\tau ,i}\;\mathrm{is}\;\mathrm{finite},\\ 0, & \mathrm{otherwise}. \end{array}\right.$$

The input validity mask follows the same definition as the target mask in Section 3.1, but it is applied to the historical input window rather than to the forecasting target.

The C = 7 configuration further adds two causal multi-scale temporal features. The 15 min causal mean is computed as

(13) $${\bar{x}}_{\tau ,i}^{15}=\mathrm{nanmean}\left\{x_{\tau -2,i},x_{\tau -1,i},x_{\tau ,i}\right\},$$

and the 60 min causal mean is computed as

(14) $${\bar{x}}_{\tau ,i}^{60}=\mathrm{nanmean}\left\{x_{\tau -11,i},\ldots ,x_{\tau ,i}\right\}.$$

For a prediction target at time t, all historical input indices satisfy

(15) τ≤t−1.

The target speed $x_{t,i}$ is never included in any causal aggregation window. If all observations within a causal window are missing, the aggregated feature remains NaN and is subsequently handled by the uniform zero-filling strategy described in Section 3.6, see Table 4.

### 3.5. TT-LSTM as a White-Box Analytical Probe

The TT-LSTM model is employed not as a competitive baseline, but as a white-box analytical probe. Its purpose is to diagnose whether hand-crafted sparse-input features provide practical gains under sparse mobile probe observations. Unlike adaptive graph neural networks, TT-LSTM does not contain adaptive graph learning or explicit graph message passing. Therefore, it provides a controlled testbed for examining the effects of topology-derived local statistics, input validity masks, and causal multi-scale temporal features.

TT-LSTM is trained region by region using the partitioned data described in Section 3.3.2. For each region, the input tensor has shape

(16) $$\left(B,L,N_{r},C\right),$$

where B is the batch size, L=12 is the lookback length, $N_{r}$ is the number of road segments in region r, and C is the number of input channels. The tensor is reshaped into

(17) $$\left(B\cdot N_{r},L,C\right),$$

So that each road segment is processed as an individual temporal sequence with shared model parameters. The sequence is first projected through a linear embedding layer. A sinusoidal positional encoding is then added to preserve temporal order. The embedded sequence is passed through a two-layer Transformer encoder to extract temporal representations. The Transformer output is then processed by a single-layer LSTM, followed by a final linear prediction head that maps the hidden representation to the next-step speed prediction. The model is intentionally kept lightweight so that the ablation focuses on input-feature effects rather than model capacity.

The overall computational flow is summarized as

(18) $$\begin{array}{cc} \left(B,L,N_{r},C\right) & \to \left(B\cdot N_{r},L,C\right)\\ & \to \mathrm{Linear}\;\mathrm{embedding}+\mathrm{positional}\;\mathrm{encoding}\\ & \to \mathrm{two-layer}\;\mathrm{Transformer}\;\mathrm{encoder}\\ & \to \mathrm{single-layer}\;\mathrm{LSTM}\\ & \to \mathrm{linear}\;\mathrm{prediction}\;\mathrm{head}\\ & \to \left(B,N_{r}\right). \end{array}$$

**Figure 3:**
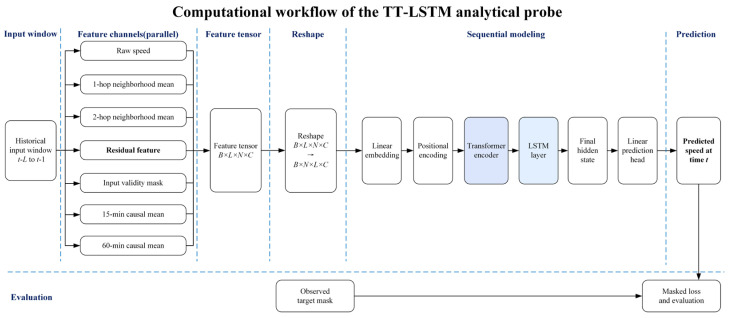
TT-LSTM computational workflow. The figure should be read together with Section 3.5: it summarizes the transformation from sparse historical feature channels to sequence embeddings, Transformer-based temporal representations, LSTM sequential modeling, and the final one-step speed prediction. This caption clarifies that TT-LSTM is used as an analytical probe for sparse-input feature effects rather than as a newly proposed state-of-the-art forecasting architecture.

Because TT-LSTM does not perform graph message passing, any difference among the C = 4, C = 5, and C = 7 configurations can be interpreted as evidence about the diagnostic value of the corresponding sparse-input feature groups, rather than as a consequence of adaptive graph learning. Figure 3 summarizes this TT-LSTM computational workflow from sparse historical feature channels to sequence embedding, Transformer encoding, LSTM sequential modeling, and one-step speed prediction. Additional TT-LSTM probe configuration details are provided in Appendix A.2.

### 3.6. Missing-History Handling for Full-Coverage Models Under Relaxed Evaluation

Under relaxed evaluation, a target position is eligible for evaluation when its ground-truth speed is directly observed and the corresponding method can generate a valid prediction under the specified support rule. The historical input window preceding that target may still contain missing values. Therefore, input missingness and target missingness are handled differently: input missingness affects the amount and reliability of temporal context available to the model, whereas target missingness determines whether a forecast error can be computed at all.

Missing historical inputs are handled uniformly across full-coverage learning models, whereas missing targets are never imputed for evaluation. Specifically, train-only z-score normalization is applied to input features. The mean and standard deviation are estimated from the training period only. After normalization, NaN values in the historical input window are uniformly filled with zeros. Because the data are normalized, a zero value represents the historical mean of the training set rather than an arbitrary physical speed.

This separation between target and input missingness is maintained throughout the study. The same principle is applied to derived features used by the GBRT baseline. For graph-based deep learning models and TT-LSTM variants, this strategy allows the model to generate predictions over all observed target positions while keeping the evaluation target mask unchanged. HIST_MEAN and DailyProfile do not require the same historical input tensor, but their prediction validity is still evaluated under the support definitions described in Section 3.8.

This design prevents missing historical observations from unnecessarily excluding observed future targets, while preserving the rule that only directly observed target speeds are used for supervised learning and metric computation.

### 3.7. Compared Methods and Train-Only Baselines

The compared methods are grouped into full-coverage models and coverage-limited baselines.

The full-coverage models include graph-based deep learning models, a tabular machine-learning baseline, a historical mean baseline, and TT-LSTM probe variants. The graph-based baselines follow the representative model families reviewed in Section 2.2 and include AGCRN, diffusion convolutional recurrent neural network (DCRNN), spatiotemporal graph convolutional network (STGCN), and GWNet. Gradient boosting regression tree (GBRT) is included as a tabular machine learning baseline using derived features. The historical mean (HIST_MEAN) baseline predicts the train-period mean speed for each road segment. TT-LSTM is evaluated under the C = 4, C = 5, and C = 7 feature configurations defined in Section 3.4.

PERSISTENCE and DailyProfile are treated as coverage-limited baselines. PERSISTENCE predicts

(19) $${\hat{x}}_{t,i}=x_{t-1,i},$$

and is valid only when $x_{t-1,i}$ is observed. PERSISTENCE requires only the immediately preceding observation and does not rely on the full 12-step lookback window. DailyProfile predicts the historical mean speed for the same road segment and time-of-day slot using training-period observations. It is valid only when the corresponding training-period slot contains sufficient observed history. Therefore, these baselines do not necessarily produce predictions for all observed target positions.

All train-only baselines are constructed without using future information from the test period. HIST_MEAN is computed from training-period observations only. DailyProfile is computed from training-period time-of-day observations only. PERSISTENCE uses only the immediately preceding observation available before the prediction target. The test period, including its future observations, is strictly excluded from the construction of all train-only baselines.

Because PERSISTENCE and DailyProfile are valid only on their own support subsets, their own-valid results are reported separately from the primary full-coverage ranking. A DailyProfile-common support is further defined in Section 3.8 to enable a sensitivity check that includes DailyProfile under a shared support condition. Detailed model training settings and hyperparameters are provided in Appendix A.1.

### 3.8. Evaluation Support Chain and Metrics

This section defines the evaluation support sets used in this paper. The exact cardinality of each support set resulting from the study dataset is provided in Section 4.1.

The full test grid is defined as

(20) $$S_{grid}=\left\{\left(t,i\right):t\in T_{test},i=1,\ldots ,N\right\}.$$

This set contains all road segment–time positions in the testing period before applying any observability constraint.

The Target-observed support is defined as

(21) $$S_{target}=\left\{\left(t,i\right)\in S_{grid}:m_{t,i}=1\right\}.$$

This support contains all positions with observed ground-truth speed and is used as the primary relaxed evaluation support throughout the primary analysis.

The Input-eligible support is defined as

(22) $$S_{input}=\left\{\left(t,i\right)\in S_{grid}:x_{t-L:t-1,i}\;\mathrm{is}\;\mathrm{complete}\right\}.$$

This set identifies positions with complete historical input for the target road segment. It is not used as the primary evaluation support by itself.

The strict Evaluation-eligible support is defined as

(23) $$S_{strict}=S_{target}\cap S_{input}.$$

This support requires both an observed target and a complete historical input window. It is used for the strict sensitivity audit.

The DailyProfile-common support is defined as

(24) $$S_{DP}=S_{target}\cap S_{DailyProfile-valid},$$

where $S_{DailyProfile-valid}$ denotes positions for which the DailyProfile baseline can produce a valid prediction from training-period observations. This support is used for the DailyProfile-common sensitivity check.

For a support set S, MAE is defined as

(25) $$MAE\left(S\right)=\frac{1}{|S|}\underset{\left(t,i\right)\in S}{\sum }|{\hat{x}}_{t,i}-x_{t,i}|.$$

RMSE is defined as

(26) $$RMSE\left(S\right)=\sqrt{\frac{1}{|S|}\underset{\left(t,i\right)\in S}{\sum }{\left({\hat{x}}_{t,i}-x_{t,i}\right)}^{2}}.$$

For congestion diagnosis, a road segment is considered congested when its speed is below 20 km/h, equivalent to approximately 5.56 m/s. This threshold is used here as the operational low-speed threshold for urban arterial congestion diagnosis. Precision, recall, and F1 score are computed using this threshold. Congestion metrics are computed on the same evaluation support as the corresponding speed metrics. This ensures that speed errors and congestion classification results are interpreted under consistent support conditions. A compact summary of the evaluation support definitions is provided in Appendix A.3.

## 4. Results

### 4.1. Evaluation Support-Chain Audit

**Figure 4:**
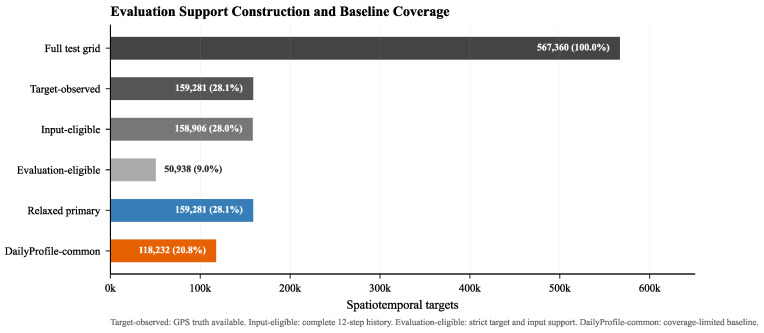
Evaluation support sets. The support-chain audit shows the contraction from the full test grid to Target-observed, strict Evaluation-eligible, relaxed primary, and DailyProfile-common supports. The relaxed primary support retains all Target-observed entries, whereas the strict complete-history criterion leaves only 9.0% of the full test grid.

The first step of the evaluation is to audit how many road segment–time targets are actually evaluable under sparse mobile probe sensing. Figure 4 and Table 5 report the support-chain contraction from the complete test grid to the observed and baseline-common evaluation supports. The full test grid contains 567,360 spatiotemporal positions. However, only 159,281 positions have observed GPS-derived target speeds, corresponding to 28.1% of the test grid. This gap between the full grid and the observed targets indicates a substantial **observability gap** caused by sparse and uneven mobile probe coverage.

The Input-eligible support has a similar marginal size to the Target-observed support, but their strict intersection is much smaller. This indicates that historical completeness and future target observability are often spatiotemporally decoupled in mobile probe sensing. In other words, a road segment may have a complete recent history but no observed target at the prediction time, or it may have an observed target but an incomplete historical input window. The contraction from 28.1% Target-observed support to 9.0% strict Evaluation-eligible support shows that requiring both observed targets and complete 12-step histories imposes a strong restriction on the evaluation support.

This support-chain audit shows that target observability, rather than model prediction alone, is the first filtering factor in sparse mobile probe evaluation. If the strict complete-history support were used as the primary evaluation setting, more than 100,000 directly observed target positions would be excluded from evaluation. Therefore, the relaxed Target-observed support is used as the primary evaluation set because it retains all directly observed ground-truth targets while allowing full-coverage models to make predictions under the unified missing-history handling protocol described in Section 3.6.

### 4.2. Primary Relaxed Evaluation on Target-Observed Support

**Figure 5:**
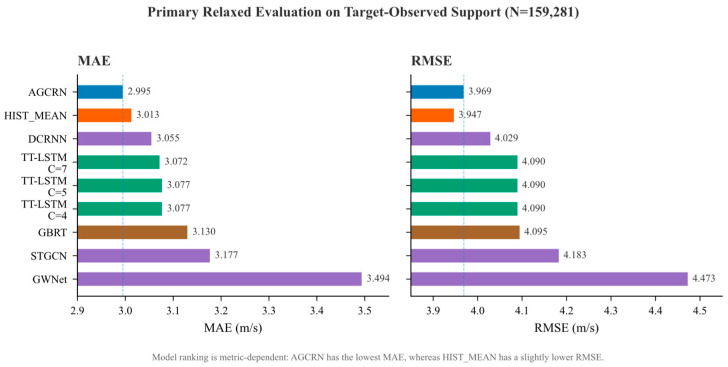
Primary relaxed evaluation on the Target-observed support. MAE and RMSE are computed on the same 159,281 observed targets. The figure highlights metric-dependent differences among models rather than a single universal winner, indicating support-conditioned and complementary strengths under sparse mobile probe sensing.

Table 6 and Figure 5 report the primary relaxed evaluation on the Target-observed support. All full-coverage models are evaluated on the same 159,281 observed road segment–time targets. This support allows a direct comparison of models without excluding targets solely because of incomplete historical inputs.

Under the primary relaxed support, the MAE and RMSE results show metric-dependent behavior rather than a single dominant model. AGCRN reduces average absolute deviations over the observed target support, whereas HIST_MEAN produces conservative predictions that limit large squared errors. This contrast indicates complementary inductive biases under ultra-short-term sparse sensing, but it should not be interpreted as universal superiority of either adaptive graph learning or historical averaging. This point is further examined in the congestion classification analysis below.

The difference between the MAE and RMSE rankings also shows that no single model dominates all evaluation dimensions. DCRNN ranks behind AGCRN and HIST_MEAN but remains competitive under both metrics. The TT-LSTM probe variants are close to each other, with C = 7 producing only a small global improvement over C = 4 and C = 5. GWNet shows larger errors under the relaxed support, suggesting that heavily learned dependency structures may be less robust in this extremely sparse setting than simpler graph-recurrent or historical-prior baselines. This interpretation is limited to the present sparse mobile probe setting and should not be generalized to all traffic forecasting benchmarks.

### 4.3. Coverage-Limited Baselines and DailyProfile-Common Sensitivity

The primary relaxed evaluation in Section 4.2 includes only full-coverage models. PERSISTENCE and DailyProfile are treated separately because their predictions are only valid when specific historical observations are available. Directly mixing their own-valid results with full-coverage rankings would confound model performance with support coverage.

#### 4.3.1. Own-Valid Baseline Results

PERSISTENCE requires the immediately preceding observation, while DailyProfile requires sufficient training-period observations for the same road segment and time-of-day slot. Their own-valid supports are therefore not random subsets of the Target-observed support. Own-valid support refers to the subset of target positions where a specific baseline can produce a valid prediction and where the corresponding ground truth is observed. Target-observed relative coverage reports the proportion of all observed target positions that remain valid for that baseline. These supports tend to concentrate on road segment–time positions with denser observations, stronger temporal continuity, and more regular historical patterns; their metrics, therefore, should be interpreted as support-conditioned results rather than direct full-coverage comparisons.

Note: Own-valid support is model-specific. Target-observed relative coverage reports the proportion of directly observed target positions that remain valid for each baseline. Metrics computed on different own-valid supports should be interpreted as support-conditioned performance rather than direct full-coverage comparisons with models evaluated on the full Target-observed support.

The lower errors of PERSISTENCE and DailyProfile on their own-valid subsets should therefore be interpreted together with their coverage limitation. Their apparent competitiveness is partly affected by a coverage penalty imposed on full-coverage models: full-coverage models must predict all observed targets, including positions lacking recent or historical support, whereas coverage-limited baselines can safely abstain from unsupported positions. In this sense, own-valid baseline results are informative about historical regularity where support exists, but they are not directly comparable with the primary full-coverage ranking.

#### 4.3.2. DailyProfile-Common Support Sensitivity

To provide a baseline-inclusive sensitivity check, the DailyProfile-common support is constructed by intersecting the Target-observed support with positions where DailyProfile can produce a valid prediction. This support contains 118,232 targets, corresponding to 20.8% of the full test grid. On this common support, DailyProfile and full-coverage models can be compared over the same evaluated road segment–time positions.

Table 7 reports the own-valid results of the coverage-limited baselines, showing that DailyProfile obtains an MAE of 3.484 m/s on its own-valid support. However, this own-valid performance is strongly shaped by coverage bias rather than predictive superiority over full-coverage models. As shown in Table 8, when evaluated on the exact same DailyProfile-common support, DailyProfile obtains an MAE of 3.484 m/s, which is higher than that of all graph-based models, TT-LSTM variants, HIST_MEAN, and GBRT.

### 4.4. TT-LSTM Probe Ablation: Sparse-Input Features and Spatial Design

**Figure 6:**
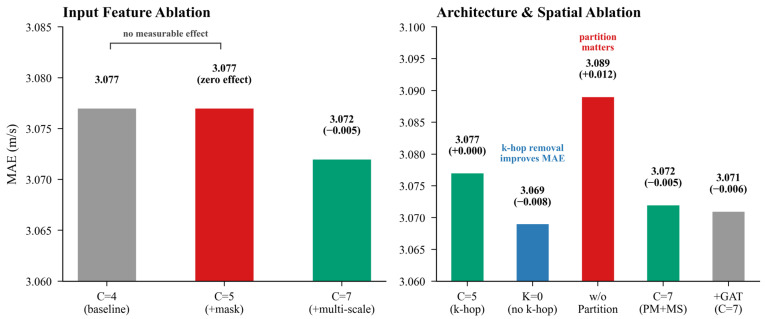
TT-LSTM probe ablation. The left panel compares C = 4, C = 5, and C = 7 input feature configurations. The right panel compares spatial and architectural variants, including k-hop removal, partition removal, C = 7 multi-scale features, and a GAT-enhanced variant. The results show limited global gains from sparse-input feature engineering and a clearer organizational benefit from partitioning.

Figure 6 presents TT-LSTM probe ablation. The left panel compares C = 4, C = 5, and C = 7 input feature configurations. The right panel compares spatial and architectural variants, including k-hop removal, partition removal, C = 7 multi-scale features, and a graph attention network (GAT)-enhanced variant. The results show limited global gains from sparse-input feature engineering and a clearer organizational benefit from partitioning.

#### 4.4.1. Input Feature Ablation

The input feature ablation compares TT-LSTM C = 4, C = 5, and C = 7 on the relaxed Target-observed support. Adding the input validity mask from C = 4 to C = 5 produces no measurable global change: both configurations obtain an MAE of approximately 3.077 m/s. This suggests that a binary missingness indicator alone is insufficient to overcome sparse input histories in this probe architecture.

Adding causal multi-scale features in C = 7 reduces the MAE to 3.072 m/s. The reduction is statistically detectable in the paired analysis reported in Section 4.8.2, but its global magnitude is operationally small. Therefore, the C = 7 configuration should be interpreted as providing smoothing information rather than a substantial accuracy gain. This small global improvement may partly reflect the reduced NaN frequency in longer causal windows, rather than a fundamentally stronger representation of traffic dynamics. A multi-seed stability check for the TT-LSTM feature ablation is provided in Appendix B.2.

#### 4.4.2. Architecture and Spatial Ablation

The architecture and spatial ablation further examines whether the local topology-derived features and regional organization contribute to the TT-LSTM probe. Removing equal-weight k-hop features does not degrade performance and may even slightly improve MAE. This null or slightly harmful effect is itself informative because it suggests that naive equal-weight spatial averaging does not usefully describe dependencies under sparse mobile probe observations.

By contrast, removing regional partitioning produces a clear degradation in MAE. This indicates that regional organization provides a more consistent benefit than naive equal-weight neighborhood averaging. The C = 7 and +GAT variants do not fundamentally alter this conclusion. The limited value of equal-weight k-hop features is also consistent with the possibility that learned adaptive graph representations, such as those used by AGCRN, are more effective under sparse mobile probe observations. Overall, the ablation suggests that the main benefit of the TT-LSTM probe comes from support-aware regional organization and causal temporal smoothing, rather than from simple equal-weight spatial averaging.

### 4.5. Full-Coverage Congestion Diagnosis

**Figure 7:**
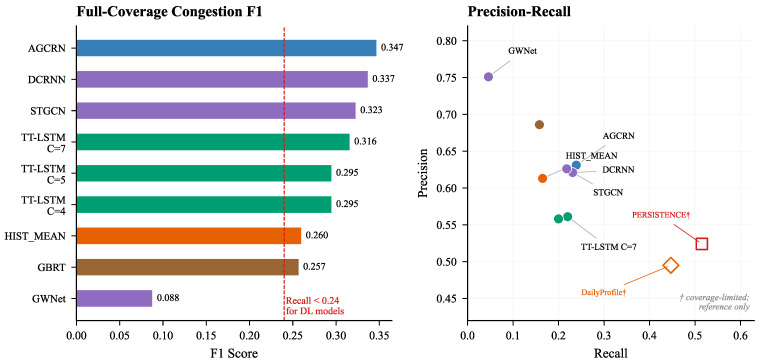
Full-coverage congestion diagnosis under the relaxed Target-observed support. The left panel reports F1 scores. The right panel reports precision–recall trade-offs. All full-coverage deep learning models exhibit recall below 0.24, indicating that congestion detection is strongly bounded by sparse observation coverage. In the precision–recall panel, filled circles denote full-coverage models, whereas open markers denote coverage-limited baselines shown for reference only.

This section evaluates whether full-coverage models can identify low-speed congested states under the same relaxed Target-observed support. A road segment–time position is classified as congested when the observed speed is below 20 km/h, equivalent to 5.56 m/s. Figure 7 presents the full-coverage congestion diagnosis results, including F1 scores and precision–recall trade-offs under the relaxed Target-observed support.

Among the full-coverage models evaluated on the same relaxed Target-observed support, AGCRN obtains the largest congestion F1 score of 0.347, followed by DCRNN and STGCN. TT-LSTM C = 7 obtains an F1 score of 0.316, slightly higher than the C = 4 and C = 5 variants. Compared with C = 4, C = 7 increases recall from 0.200 to 0.220 and F1 from 0.295 to 0.316. This suggests that multi-scale smoothing provides limited robustness for low-speed states, but the improvement remains modest and should not be interpreted as accurate full-coverage congestion detection, see Table 9.

The precision–recall analysis shows that all full-coverage deep learning models have recall below 0.24. This indicates that congestion detection under sparse probe sensing is strongly bounded by observation coverage rather than by model capacity alone. HIST_MEAN illustrates the complementary trade-off discussed in Section 4.2. Although it has the smallest RMSE in the primary speed evaluation, its congestion recall is only 0.165, indicating limited sensitivity to low-speed state transitions.

Coverage-limited baselines may achieve higher own-valid recall where historical support exists. For example, PERSISTENCE achieves a recall of 0.515 on its own-valid subset. This suggests that periodic or continuous congestion is detectable where recent support exists. However, full-coverage congestion detection remains constrained when all observed targets are included, especially at positions without recent or dense historical observations.

GWNet’s unusually low F1, together with its higher overall speed error and congestion MAE, suggests that its predictions fail to capture low-speed states in this sparse sensing setting. This observation should be interpreted as a result of the present sparse-probe evaluation condition rather than as a general statement about the model family.

### 4.6. Speed-Bin Error Diagnosis

**Figure 8:**
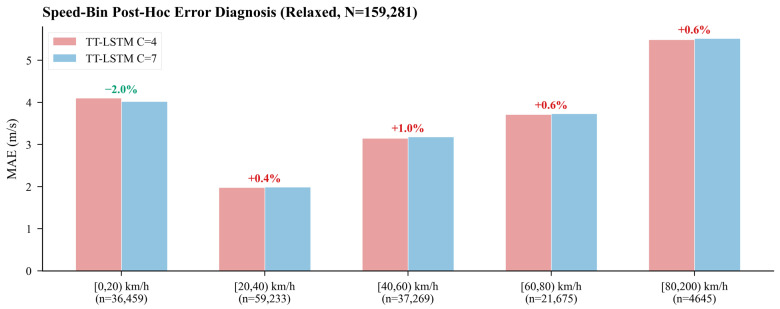
Speed-bin post hoc error diagnosis for TT-LSTM C = 4 and C = 7 under the relaxed Target-observed support. Bins are defined by observed target speed. Multi-scale features reduce MAE in the low-speed bin but slightly increase MAE in several medium- and high-speed bins, explaining why the global gain is small.

The previous ablation shows that TT-LSTM C = 7 provides only a small global MAE reduction compared with C = 4. To understand where this reduction occurs, Figure 8 reports a post hoc speed-bin error diagnosis conditioned on the observed target speed.

The observed targets are grouped into five speed bins: [0, 20), [20, 40), [40, 60), [60, 80), and [80, 200) km/h. The lowest-speed bin contains 36,459 targets and corresponds to congested or near-congested conditions. In this bin, TT-LSTM C = 7 reduces MAE relative to C = 4 by 0.082 m/s, equivalent to a reduction of approximately 2.0%. In contrast, the [40, 60), [60, 80), and [80, 200) km/h bins show slight MAE increases of 0.032, 0.021, and 0.031 m/s, respectively.

This pattern indicates that the small global improvement of C = 7 is not uniform across traffic regimes. The causal multi-scale features are more useful in low-speed conditions, where they may reduce noisy fluctuations caused by sparse input histories, but this benefit is diluted or offset in more frequent medium-speed regimes. Because speed-bin diagnostics are conditioned on true observed speed, these patterns reflect post hoc model behavior across traffic regimes rather than online congestion prediction skill. The low-speed improvement of C = 7 may partly come from alleviating missing historical inputs through longer causal windows, rather than from deep modeling of congestion dynamics.

### 4.7. Partition-Boundary Diagnosis

**Figure 9:**
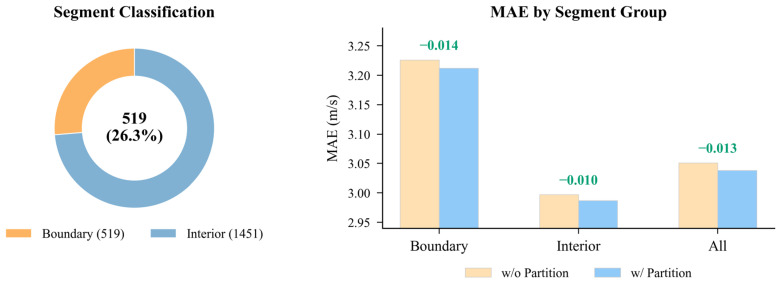
Partition-boundary diagnosis. The left panel reports the proportion of boundary and interior segments. The right panel compares MAE for boundary, interior, and all segments with and without partitioning. Partitioning yields small but consistent MAE reductions for both boundary and interior segments, indicating that it does not introduce a boundary penalty.

Regional partitioning is used as an engineering organization strategy, but it may raise a concern that boundary segments could suffer from partition-induced discontinuities. Figure 9 examines this possibility by comparing boundary and interior road segments.

The road segments are classified into 519 boundary segments and 1451 interior segments. Boundary segments account for 26.3% of the evaluated road segments. The MAE comparison shows that partitioning reduces MAE for boundary, interior, and all segments. The reductions are small but consistent, with approximate decreases of 0.014 m/s for boundary segments, 0.010 m/s for interior segments, and 0.013 m/s overall.

Partitioning, therefore, does not introduce a boundary penalty. Instead, it yields small but consistent MAE reductions for both boundary and interior segments. Boundary segments remain harder than interior segments in absolute error, with boundary errors approximately 7.6% higher than interior errors. This primarily reflects their more complex or sparse topological context rather than a partition-induced artifact.

### 4.8. Strict Evaluation Sensitivity and Representativeness Audit

The final part of the Results section examines the relationship between strict and relaxed evaluation. The purpose is not to reject strict evaluation but to show that strict complete-history filtering evaluates a narrower and more selective subset of the observed targets.

#### 4.8.1. Strict vs. Relaxed Model Comparison

**Figure 10:**
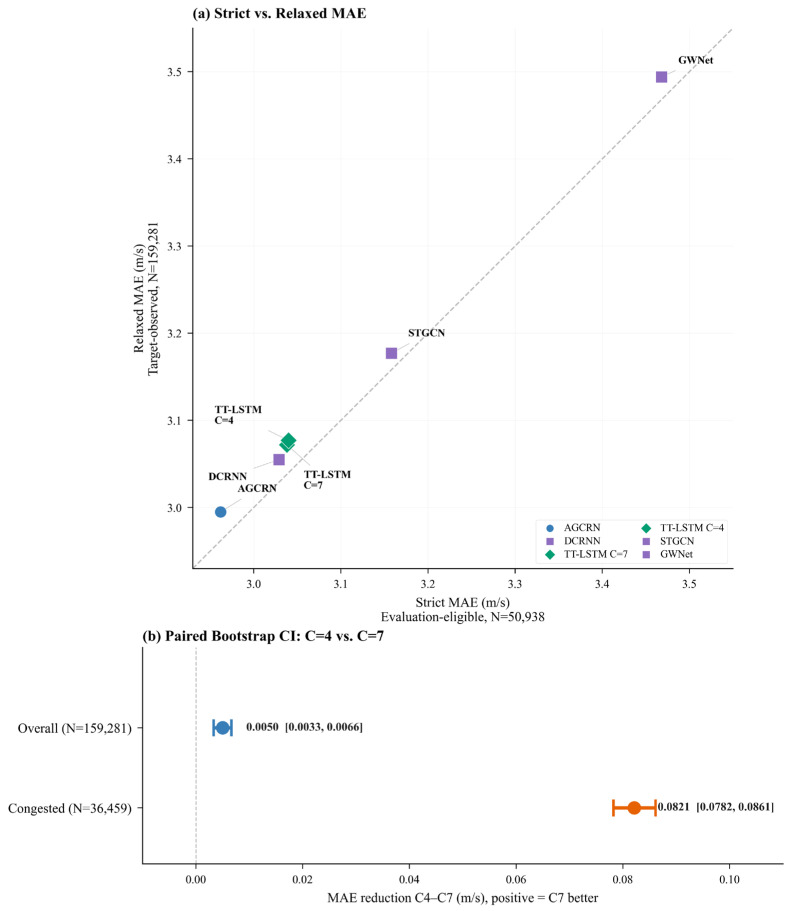
Strict evaluation sensitivity and paired bootstrap analysis. The strict-vs-relaxed scatter plot shows that relaxed evaluation is slightly harder while preserving the broad ranking pattern. The paired bootstrap panel shows that C = 7 yields a statistically detectable but operationally small global MAE reduction compared with C = 4, with a larger reduction in the congested subset.

Figure 10 compares model MAE under the strict Evaluation-eligible support and the relaxed Target-observed support. The strict support contains 50,938 targets, whereas the relaxed support contains 159,281 targets.

Most points lie above the diagonal, indicating that relaxed evaluation is slightly harder than strict evaluation. At the same time, model ranking is largely preserved. This suggests that strict evaluation is not misleading in ranking direction, but it evaluates a much narrower and more selective subset of the observed targets. The relaxed support better reflects all directly observed targets available in the test period. The exact numerical MAE values under the strict Evaluation-eligible support are provided in Appendix B.1.

#### 4.8.2. Paired Bootstrap for TT-LSTM C = 4 vs. C = 7

A paired bootstrap analysis is conducted to quantify the MAE reduction of TT-LSTM C = 7 relative to C = 4. On the overall relaxed support, C = 7 reduces MAE by 0.0050 m/s, with a 95% confidence interval of [0.0033, 0.0066]. The confidence interval excludes zero, indicating that the reduction is statistically detectable. However, the global magnitude remains operationally small.

On the congested subset, the reduction is larger: 0.0821 m/s, with a 95% confidence interval of [0.0782, 0.0861]. This confirms that the causal multi-scale features are more useful under low-speed conditions. However, the congested subset accounts for only 36,459 targets, or approximately 22.9% of the relaxed support, and the improvement in this subset is partly offset by slight degradation in medium- and high-speed bins. This explains why the global gain remains small.

#### 4.8.3. Strict Subset Representativeness Audit

**Figure 11:**
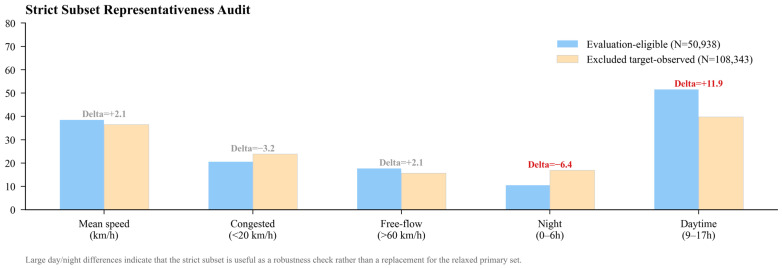
Strict subset representativeness audit. The strict Evaluation-eligible support and the excluded Target-observed positions differ in mean speed, congestion proportion, night-time representation, and daytime representation. The strict subset under-represents night-time and congested conditions and over-represents observation-rich daytime positions. These imbalances support treating strict evaluation as a sensitivity analysis rather than as the primary ranking support.

Figure 11 compares the strict Evaluation-eligible subset with the Target-observed positions excluded by the strict complete-history requirement. This analysis examines the representative bias of strict evaluation.

The strict complete-history subset differs from the excluded Target-observed positions in both temporal and traffic-state composition. The strict subset has a mean speed of 38.6 km/h, compared with 36.5 km/h for the excluded positions. It also has a lower congestion proportion: 20.7% versus 23.9%. Temporally, night-time positions account for 10.6% of the strict subset but 17.0% of the excluded positions, while daytime positions account for 51.7% of the strict subset but 39.8% of the excluded positions.

These differences show that the strict subset under-represents night-time positions by 6.4 percentage points and congested states by 3.2 percentage points, while over-representing daytime positions by 11.9 percentage points. This bias is not catastrophic, but it is large enough to caution against directly generalizing strict evaluation results to the entire Target-observed support. The non-negligible temporal and traffic-state imbalance of the strict subset further supports the choice of Target-observed support as the primary evaluation set.

The support-chain audit is therefore not only a fairness check but also an integrity check on whether the evaluated targets represent the sparse mobile sensing observations available in the test period. This closes the evaluation loop: relaxed evaluation retains all directly observed targets, while strict evaluation is best treated as a sensitivity analysis rather than the primary ranking criterion.

## 5. Discussion

### 5.1. Evaluation Support as a First-Order Experimental Factor

The results show that, under sparse mobile probe sensing, the evaluation support defines the empirical question being answered—model accuracy, baseline competitiveness, and congestion diagnosis can only be interpreted after this support is made explicit. In conventional fixed-sensor forecasting settings, the test grid is often implicitly treated as the evaluable target set. In contrast, mobile probe sensing creates an illusion of full coverage: the road network and test period define a large spatiotemporal grid, but only a fraction of these positions have directly observed GPS-derived ground truth. The support-chain audit makes this hidden unobservability explicit, treating sparse sensing not as a data defect to be imputed away, but as a defining constraint on what can be empirically verified.

This distinction is not a minor implementation detail. A model evaluated on all observed targets and a baseline evaluated only on observation-rich positions are not answering the same empirical question. Under sparse mobile probe sensing, failing to report evaluation support can produce an artificial sense of precision in model comparisons, because the compared models may be evaluated on different subsets of the data. This is especially important when full-coverage models are compared with coverage-limited baselines such as PERSISTENCE or DailyProfile. The former are required to generate predictions for all observed targets, including weakly supported positions, whereas the latter are only evaluated where their required historical observations exist.

The strict Evaluation-eligible subset provides a useful sensitivity check, but it should not automatically become the default primary benchmark setting. Its complete-history requirement yields a smaller and more selective subset of observed targets. As shown in the representativeness audit, this subset under-represents night-time and congested conditions while over-representing daytime observation-rich positions. This does not invalidate strict evaluation, but it clarifies that strict evaluation should be treated as a sensitivity analysis rather than as the default primary benchmark setting—a distinction that the support-chain audit is designed to make explicit rather than leaving as an implicit assumption.

Therefore, the primary implication of this study is methodological: evaluation support should be treated as a first-order experimental factor. For sparse-probe traffic forecasting, reporting MAE or RMSE without reporting the corresponding support definition, support size, and model coverage is insufficient. The mask-consistent relaxed Target-observed support is adopted in this paper because it retains all directly observed ground-truth targets and better represents the true, operationally verifiable scope of the sensing system.

### 5.2. Complementary Roles of Adaptive Graph Learning and Historical Priors

The primary relaxed evaluation reveals a complementary relationship between adaptive graph learning and historical priors. AGCRN yields the smallest MAE, while HIST_MEAN yields the smallest RMSE on the primary relaxed Target-observed support. This difference should not be interpreted as a contradiction or as a simple winner-takes-all ranking. Instead, it reflects two different inductive biases under sparse ultra-short-term forecasting.

AGCRN introduces a spatial structural inductive bias through adaptive graph learning. This allows the model to use learned spatial dependencies beyond predefined adjacency to reduce average absolute deviations over the observed target support. However, under ultra-short-term sparse probe sensing, this advantage is conditional: limited temporal observations, unstable target support, and weak recurrent evidence for rarely observed segments can reduce the benefit of adaptive graph learning. In contrast, HIST_MEAN imposes a conservative temporal periodic inductive bias. By predicting a train-period historical average, it avoids extreme deviations and therefore performs well under RMSE, a metric that penalizes large errors more strongly.

However, this RMSE advantage comes with a trade-off. HIST_MEAN is less sensitive to traffic state transitions, especially low-speed transitions associated with congestion. Its congestion recall is only 0.165, substantially lower than AGCRN’s 0.239. This shows that a low RMSE does not necessarily imply strong traffic-state recognition. Under sparse sensing, conservative predictions can be numerically stable yet operationally blind to the low-speed events that matter most for traffic management.

The divergence between the models favored by MAE and RMSE indicates that sparse-probe forecasting cannot be adequately summarized by a single accuracy metric. Because MAE, RMSE, and congestion-oriented indicators reflect fundamentally different aspects of forecasting quality, this contrast cautions against single-metric benchmarking under sparse sensing. Instead, it favors multi-faceted reporting that includes both error metrics and task-sensitive diagnostics such as congestion recall, always together with the evaluation support on which they are computed.

### 5.3. Why Simple Sparse-Input Feature Engineering Provides Limited Global Gains

The TT-LSTM probe results show that sparse-input feature engineering is not automatically beneficial. The usefulness of an added feature depends on whether it captures the observation mechanism, rather than merely increasing the number of input channels. This is why the input validity mask produces almost no measurable global effect, while causal multi-scale features provide only a small overall improvement.

The binary input validity mask creates an information bottleneck. It tells the model whether an observation exists, but it does not encode how long the segment has been unobserved, how many probe vehicles contributed to the observation, how reliable the observed speed is, or how dense the surrounding sensing context may be. Under sparse mobile probe sensing, these missing details can be more important than the binary distinction between observed and unobserved inputs. In effect, the mask encodes the presence of an observation but not the quality, density, or recency of the sensing context—information that may be more diagnostic than the binary flag itself.

The equal-weight k-hop statistics also show limited value. Such features assume that neighboring road segments contribute uniformly, but real traffic dependencies are shaped by directionality, road class, signal control, demand distribution, turning movements, and local network structure. Under sparse observations, equal-weight averaging can even propagate weak or noisy information. The limited value of these hand-crafted local averages is consistent with the stronger performance of adaptive graph models, which can learn more flexible spatial dependencies from data.

The C = 7 configuration provides a small global improvement, but the speed-bin diagnosis shows that this benefit is concentrated in low-speed conditions. This suggests that causal multi-scale features act more as a low-speed smoothing aid than as a general accuracy amplifier. Moreover, because longer causal windows reduce the frequency of entirely missing aggregated values, part of this low-speed benefit may reflect mitigated input sparsity rather than deeper causal modeling of congestion dynamics.

These findings suggest that future sparse-input designs should move beyond simple masks and equal-weight averages. More promising directions include probe-count-aware features, uncertainty-aware input reliability, learned spatial dependencies, and temporal encodings that explicitly represent observation gaps. However, these directions remain hypotheses to be tested under the same support-chain audit framework introduced here, rather than conclusions of the current study.

### 5.4. Congestion Diagnosis Under Sparse Mobile Probe Sensing

The congestion results indicate that low recall is not merely a model-specific failure, but a manifestation of limited observation support under mobile probe sensing. Although AGCRN obtains the largest full-coverage congestion F1 score in this evaluation, all full-coverage deep learning models exhibit recall below 0.24. This pattern suggests that the central difficulty lies not only in model capacity, but also in the sparse and uneven availability of informative historical observations for low-speed states.

Coverage-limited baselines provide an important contrast. They may achieve higher recall on their own-valid subsets, which confirms that recurrent or periodic congestion patterns exist and are learnable where historical support is sufficient. However, these own-valid subsets are not the same as the full Target-observed support. They tend to include positions with recent or repeated observations, where temporal continuity is stronger and congestion is easier to infer.

The difficulty lies in extending congestion detection to the full set of observed targets, many of which lack the dense temporal context that makes periodicity-based detection possible. In other words, coverage-limited baselines can detect recurrent congestion where they have enough historical support, but full-coverage models must also make predictions in weakly supported spatiotemporal voids, where both recurrent patterns may be absent and non-recurrent events are harder to distinguish from noise. The low recall implies that spotting congestion in unsensed or weakly supported parts of the network remains a systemic barrier, not just a modeling shortcoming.

This result has practical implications. Improving full-coverage congestion recall may require denser sensing inputs, complementary sensing modalities, or explicit reliability features, rather than merely increasing model depth or architectural complexity. Incorporating probe counts, observation recency, fixed-sensor data, signal timing, incident reports, or weather information may help distinguish true low-speed states from uncertainty caused by sparse observations. Without such additional support, full-coverage congestion diagnosis from sparse mobile probes alone remains intrinsically difficult, as the present audit demonstrates, rather than a limitation that can be engineered away by deeper architectures alone.

### 5.5. Implications for Benchmarking and Deployment

The findings have direct implications for benchmarking. A benchmark under sparse mobile sensing should specify not only what model is evaluated, but also where it is evaluated. Support size, support definition, model coverage, and baseline validity conditions should be reported alongside MAE, RMSE, and congestion metrics. Otherwise, the apparent ranking of models may reflect support differences rather than modeling capability.

This is particularly important when comparing full-coverage models with coverage-limited baselines. Own-valid results are useful because they reveal how well a baseline performs where its assumptions are satisfied. However, they should not be mixed directly with full-coverage rankings. The present audit suggests that a transparent sparse-probe evaluation should include at least three complementary views: primary all-observed evaluation, own-valid baseline evaluation, and common-support sensitivity checks. Strict complete-history evaluation can also be reported, but it should be framed as a sensitivity analysis rather than as the only benchmark setting.

The findings also have implications for deployment. In practical traffic management systems, a predicted speed should not be treated as equally reliable everywhere. A prediction produced with complete recent observations is different from a prediction produced after extensive zero-filling of missing inputs. Therefore, a deployed system should ideally output not only predicted speed, but also the sensing support condition behind that prediction. This may include recent observation availability, historical coverage, probe count, or a confidence flag.

This points toward confidence-calibrated forecasting. For human-in-the-loop traffic management, a prediction accompanied by its support status allows operators to distinguish between high-confidence estimates and unsupported extrapolations. This can reduce the risk of over-reliance on forecasts in data-sparse regions, for example, preventing a false alarm from triggering unnecessary dynamic speed limits simply because of a zero-filled input window. In this sense, support-chain auditing bridges the gap between research benchmarking and operational transparency, providing a common language for communicating where and when a prediction can be trusted.

### 5.6. Limitations and Future Work

This study has several limitations. First, the dataset covers only three consecutive days. This period provides sufficient sparsity and short-term temporal variability for a controlled support-chain audit, but it is not sufficient for drawing universal conclusions about long-term traffic forecasting. Weekday/weekend effects, incident-specific fluctuations, seasonal variations, holiday effects, spatial transferability, and long-term demand shifts cannot be fully evaluated with the available data. Longer observation periods, additional cities, and richer sensing conditions are needed to test whether the same support-chain behavior holds under broader temporal and spatial conditions.

Second, the analysis is based on one urban road network and one mobile probe data source. The results may depend on local road structure, ride-hailing demand distribution, road hierarchy, and probe penetration. Future work should test the same audit protocol across multiple cities, different network morphologies, and different sensing densities.

Third, this study uses deterministic nearest-segment spatial assignment rather than a full probabilistic map-matching procedure. This simplifies trajectory-road association and may introduce spatial assignment noise in dense intersections or complex road geometries. The 30 m threshold effectively reduces gross mismatches, keeping the analytical focus on evaluation support rather than map-matching optimization. In dense urban areas, this approach may also confuse parallel links, turning movements near intersections, or low-speed/idling vehicles waiting at traffic signals with congestion on adjacent segments. Because lane-level trajectories, signal phase information, and high-resolution map-matching probabilities were not available, the present study treats such assignment uncertainty as a data-preprocessing limitation rather than as a separately modeled error source. Nevertheless, future studies could explicitly examine how map-matching uncertainty interacts with support-chain evaluation.

Fourth, this study relies on ride-hailing GPS observations and does not incorporate fixed detectors, signal timing, weather, incidents, road capacity, or other external data sources. This limitation is especially relevant for congestion diagnosis, where low recall suggests that sparse mobile probe data alone may not provide enough information for robust full-coverage detection. Multi-source sensing and fusion are natural extensions.

Finally, TT-LSTM is used as a white-box analytical probe rather than as a final forecasting architecture. Its role is to diagnose the effects of input masks, k-hop statistics, causal multi-scale features, and regional organization. Therefore, the limited gains observed from TT-LSTM feature engineering should be interpreted as diagnostic evidence about simple sparse-input designs, not as an upper bound on future support-aware forecasting architectures.

Within these boundaries, the present study establishes a principled starting point for treating evaluation support as a first-class experimental variable—a dimension that has been largely implicit in prior sparse-probe forecasting research. Future work should extend this mask-consistent audit protocol to longer multi-city datasets and richer sensing modalities. On the modeling front, it encourages the development of support-aware graph neural networks and uncertainty-guided architectures that explicitly encode observation reliability.

## 6. Conclusions

This paper investigated road-segment speed forecasting under sparse mobile probe sensing—specifically, ride-hailing GPS trajectories that produce incomplete, uneven, and spatially biased observations. The central objective was not only to compare forecasting models but to audit how target observability, input eligibility, prediction coverage, and baseline validity collectively shape model evaluation.

The support-chain audit showed that only 159,281 of the 567,360 positions in the full test grid were directly observed, and imposing a strict complete-history criterion further reduced the evaluable support to 50,938 positions. This contraction demonstrates that sparse sensing turns the evaluation set itself into an experimental variable—one that must be explicitly reported alongside model accuracy, not assumed away. Consequently, the relaxed Target-observed support was adopted as the primary evaluation set because it retains all verifiable ground-truth targets.

On the primary relaxed support, the results showed metric-dependent and support-conditioned behavior: AGCRN reduced MAE, whereas HIST_MEAN reduced RMSE. This should be interpreted as complementary evidence about adaptive graph learning and conservative historical priors under this controlled sparse-sensing case study, not as a universal ranking across all traffic forecasting settings. The separate analysis of PERSISTENCE and DailyProfile further showed that coverage-limited baselines must be interpreted together with their own-valid supports, because mixing them with full-coverage rankings introduces coverage bias.

The TT-LSTM white-box analytical probe further revealed that common sparse-input feature engineering provides limited global benefit in this setting. The input validity mask had no measurable effect, equal-weight k-hop averaging was not beneficial, and causal multi-scale features produced only a small global gain while moderately improving low-speed errors. In addition, all full-coverage deep learning models exhibited low congestion recall, indicating that congestion diagnosis under sparse mobile probe sensing is bounded by observation coverage rather than by model capacity alone.

The main methodological implication of this study is that sparse-probe forecasting benchmarks should report not only what model is evaluated, but also exactly where it is evaluated. Metrics computed on different supports answer fundamentally different empirical questions and should not be interpreted as directly comparable. Making the evaluation support explicit is, therefore, a prerequisite for trustworthy model comparison and congestion diagnosis under sparse mobile sensing.

Future work should extend the mask-consistent support-chain audit to longer multi-city datasets, richer sensing modalities, weekday/weekend and incident-aware validation, and forecasting architectures that explicitly encode observation reliability. In this way, evaluation support can move from a post hoc diagnostic object to a design principle—one that shapes how data are collected, how models are trained, how prediction confidence is communicated, and how forecasts are deployed.

## Figures and Tables

**Table 1:** Nomenclature of key symbols and support definitions.

Category	Symbol/Term	Meaning
Matrix and mask notation	$X\in {\left(\mathbb{R}\cup \left\{\mathrm{NaN}\right\}\right)}^{T\times N}$	Road-segment speed observation matrix, where missing segment-time observations are represented as NaN.
	$x_{t,i},{\hat{x}}_{t,i}$	Observed and predicted speeds of road segment *i* at time step t, respectively.
	T, N	Numbers of time steps and road segments, respectively.
	L, H	Historical look-back length and forecasting horizon.
	$M^{obs}$	Observation-validity mask, where $m_{t,i}^{obs}=1$ if $x_{t,i}$ is finite and $m_{t,i}^{obs}=0$ otherwise.
	$M^{pred}$	Prediction-validity mask indicating whether a method can produce a valid prediction at a segment-time position.
Evaluation support set	$S_{grid}$	Full test grid, including all road segment-time positions in the test period.
	$S_{target}$	Target-observed support, including positions where the ground-truth target speed is directly observed.
	$S_{strict}$	Strict Evaluation-eligible support, including positions in S_{target} with complete 12-step historical input support.
	$S_{own}^{\left(k\right)}$	Own-valid subset for method k, including positions where method k can produce valid predictions and the target is observed.
	$S_{common}$	Common-support subset shared by the compared methods.
	Mask-consistent evaluation	Evaluation in which metrics are computed only on positions satisfying the specified observation, prediction-validity, and support constraints.
	Relaxed primary evaluation	Primary evaluation on S_{target}, under the specified prediction-validity rule for each full-coverage model.
	Coverage relative to $S_{target}$	Proportion of Target-observed positions with valid predictions, i.e., the number of valid predictions divided by the number of Target-observed positions
Missingness term	Input missingness	Missing observations within the L = 12 historical input window.
	Target missingness	Missing ground-truth speed at the prediction target position.

**Table 2:** Summary of related studies on traffic speed forecasting under sparse sensing conditions.

Study Group	Representative Methods	Data Source	Missingness Treatment	Evaluation Limitation
Deep temporal models	LSTM, GRU	Loop detectors or FCD	Imputation or complete-grid filtering	Often assume stable observation support
Graph neural models	STGCN, DCRNN, AGCRN	Sensor or road networks	Spatial propagation or learned adjacency	Sparse target validity is rarely audited
Transformer-based models	PDFormer, STAEformer	Multi-source traffic data	Temporal attention over historical sequences	Support inconsistency is often implicit
Sparse probe studies	Taxi GPS, ride-hailing GPS, FCD	Mobile probes	Aggregation, filtering, map matching	Cross-model comparison may be support-biased
This paper	Mask-consistent support-chain audit	Ride-hailing GPS	Missing targets not imputed for evaluation	-

**Table 3:** Training and testing settings used in the forecasting experiments.

Item	Setting Used in the Code	Source/Note
Temporal resolution	5 min	Configs/experiment.json; speed matrix aggregation
Observation period	17 October 2019 to 19 October 2019 (864 time slots)	3-day period
Train/test split	576 train slots/288 test slots	First two days for training, final day for testing
Forecasting task	Single-step road-segment speed prediction	Horizon = 1, next 5 min step
Look-back window	12 time steps (60 min)	Historical input window
Road-segment organization	22 spatially coherent regions	Section 3.3
Partition method	non-negative matrix factorization (NMF) (K = 12) + Snake clustering	Section 3.3
Primary evaluation support	Target-observed support	Only directly observed targets enter metrics
Sensitivity supports	Strict and common-support subsets	Used for support-chain audit
Missing target handling	Targets are never imputed for metrics	Mask-consistent evaluation
Missing input handling	Train-only z-score normalization; NaN historical inputs filled with 0 after normalization	Missing targets are not imputed for metrics
Graph model settings	See Appendix A	Optimizer, batch size, epochs, etc.
TT-LSTM settings	See Appendix A	Architecture and training details
Metrics	MAE, RMSE, congestion precision/recall/F1	Mask-consistent evaluation

**Table 4:** Input feature configurations for the TT-LSTM probe model.

Configuration	Channels	Description
C = 4	4	Raw speed, 1-hop mean, 2-hop mean, residual
C = 5	5	C = 4 + input validity mask
C = 7	7	C = 5 + 15 min and 60 min causal means

**Table 5:** Evaluation support set counts.

Support Set	Number of Targets	Percentage of Full Test Grid	Interpretation
Full test grid	567,360	100.0%	All road segment–time positions in the test period
Target-observed	159,281	28.1%	GPS-derived target truth available
Input-eligible	158,906	28.0%	12-step historical input structurally available
Strict Evaluation-eligible	50,938	9.0%	Observed target and complete 12-step history
Relaxed primary	159,281	28.1%	All directly observed targets
DailyProfile-common	118,232	20.8%	Support shared with the DailyProfile baseline

**Table 6:** Primary relaxed model comparison on the Target-observed support.

Model	Evaluation Support	Coverage	MAE (m/s)	RMSE (m/s)	MAE Rank	RMSE Rank
AGCRN	159,281	100.0%	2.995	3.969	1	2
HIST_MEAN	159,281	100.0%	3.013	3.947	2	1
DCRNN	159,281	100.0%	3.055	4.029	3	3
TT-LSTM C = 7	159,281	100.0%	3.072	4.090	4	4–6
TT-LSTM C = 5	159,281	100.0%	3.077	4.090	5–6	4–6
TT-LSTM C = 4	159,281	100.0%	3.077	4.090	5–6	4–6
GBRT	159,281	100.0%	3.130	4.095	7	7
STGCN	159,281	100.0%	3.177	4.183	8	8
GWNet	159,281	100.0%	3.494	4.473	9	9

**Table 7:** Own-valid results of coverage-limited baselines.

Baseline	Own-Valid Support	Coverage Relative Target-Observed	MAE (m/s)	RMSE (m/s)	Note
PERSISTENCE	92,857	58.3%	3.342	4.625	Requires $x_{t-1,i}$ observed
DailyProfile	118,232	74.2%	3.484	4.742	Required train-period slot history

**Table 8:** DailyProfile-common support sensitivity.

Model	Evaluation Support	MAE(m/s)	RMSE(m/s)	Note
AGCRN	118,232	2.909	3.868	Full-coverage model evaluated on $S_{DP}$
DCRNN	118,232	2.954	3.914	Full-coverage model evaluated on $S_{DP}$
TT-LSTM C = 7	118,232	2.954	3.899	Probe model evaluated on $S_{DP}$
TT-LSTM C = 5	118,232	2.962	3.903	Probe model evaluated on $S_{DP}$
TT-LSTM C = 4	118,232	2.962	3.903	Probe model evaluated on $S_{DP}$
HIST_MEAN	118,232	2.965	3.881	Full-coverage baseline evaluated on $S_{DP}$
GBRT	118,232	3.025	3.973	Tabular baseline evaluated on $S_{DP}$
STGCN	118,232	3.078	4.069	Full-coverage model evaluated on $S_{DP}$
GWNet	118,232	3.397	4.363	Full-coverage model evaluated on $S_{DP}$
DailyProfile	118,232	3.484	4.742	Coverage-limited baseline on common support

**Table 9:** Full-coverage congestion classification metrics.

Model	Precision	Recall	F1 Score	Congestion MAE (m/s)
AGCRN	0.631	0.239	0.347	3.74
DCRNN	0.621	0.231	0.337	3.83
STGCN	0.626	0.218	0.323	3.95
TT-LSTM C = 7	0.561	0.220	0.316	4.02
TT-LSTM C = 5	0.558	0.200	0.295	4.11
TT-LSTM C = 4	0.558	0.200	0.295	4.11
HIST_MEAN	0.613	0.165	0.260	4.14
GBRT	0.686	0.158	0.257	4.16
GWNet	0.751	0.046	0.088	4.91

## Data Availability

The traffic speed dataset used in this study is publicly available from Lifang Data Society. OpenStreetMap road network data are publicly available from OpenStreetMap. The derived experimental results, evaluation masks, and support-chain audit outputs generated in this study are available from the corresponding author upon reasonable request.

## Abbreviations

The following abbreviations are used in this manuscript:

AGCRN	Adaptive Graph Convolutional Recurrent Network
CI	Confidence Interval
DCRNN	Diffusion Convolutional Recurrent Neural Network
GAT	Graph Attention Network
GBRT	Gradient Boosting Regression Tree
GCN	Graph Convolutional Network
GNN	Graph Neural Network
GWNet	Graph WaveNet
HIST_MEAN	Historical Mean
LSTM	Long Short-Term Memory
MAE	Mean Absolute Error
RMSE	Root Mean Squared Error
NMF	Non-negative Matrix Factorization
NNDSVDa	Non-negative Double Singular Value Decomposition with Averaging
OSM	OpenStreetMap
PR	Precision–Recall
STGCN	Spatio-Temporal Graph Convolutional Network
TT-LSTM	Transformer–LSTM
UTM	Universal Transverse Mercator
D2STGNN	Decoupled Dynamic Spatial–Temporal Graph Neural Network
PDFormer	Propagation Delay-aware Dynamic Long-range Transformer
STAEformer	Spatiotemporal Adaptive Embedding Transformer
STID	Spatial–Temporal Identity
STGym	Spatiotemporal Gym
TFB	Time Series Forecasting Benchmark
TSI-Bench	Time Series Imputation Benchmark

## Appendix A

### Appendix A.1. Model Training Settings

Table A1 summarizes the main training settings used for the forecasting models. The lookback window was fixed at L = 12, corresponding to 60 min of historical observations, and the prediction horizon was fixed at H = 1, corresponding to the next 5 min time step.

**Table A1:** Model training settings.

Model	Learning Rate	Batch Size	Max Epochs	Lookback	Horizon	Device
AGCRN	1 × 10^−3^	32	50	12	1	Apple MPS
DCRNN	1 × 10^−3^	32	50	12	1	Apple MPS
STGCN	1 × 10^−3^	32	50	12	1	Apple MPS
GWNet	1 × 10^−3^	16	50	12	1	Apple MPS
TT-LSTM	1 × 10^−3^	256	50	12	1	Apple MPS
GBRT	-	-	-	12	1	CPU
HIST_MEAN	-	-	-	-	1	CPU
DailyProfile	-	-	-	-	1	CPU
PERSISTENCE	-	-	-	-	1	CPU

Note: Deep learning models (AGCRN, DCRNN, STGCN, GWNet, and TT-LSTM) were implemented using PyTorch 2.9 (Meta Platforms, Inc., Menlo Park, CA, USA) and trained on Apple Metal Performance Shaders (MPS; Apple Inc., Cupertino, CA, USA). The GBRT baseline was implemented using XGBoost (xgboost 2.x). Data processing and analysis relied on Python 3.9 (Python Software Foundation, Wilmington, DE, USA) with NumPy, SciPy, pandas, scikit-learn, NetworkX, Matplotlib, GeoPandas, and Shapely. Non-parametric or rule-based baselines (HIST_MEAN, DailyProfile, PERSISTENCE) were evaluated on CPU and do not require learning rate, batch size, or epoch settings.

### Appendix A.2. TT-LSTM Probe Configuration

Table A2 reports the detailed configuration of the TT-LSTM white-box analytical probe. The model was designed as a lightweight diagnostic model rather than as a final forecasting architecture.

**Table A2:** TT-LSTM probe configuration.

Component	Setting
Input shape	$\left(B,\;L,\;Nr\;,\;C\right)$
Lookback length	L=12
Horizon	H=1
Feature settings	C = 4,5,7
Linear embedding	Input channels $C\to d_{model}=64$
Positional encoding	Sinusoidal positional encoding
Transformer encoder	2 layers
Attention heads	4
Feed-forward dimension	$d_{ff}=128$
Activation	GELU
Recurrent layer	1-layer LSTM
LSTM hidden size	64
Training unit	Region-wise
Number of regions	22
No-partition control	All road segments merged into one pseudo-region
Parameters per region	Approximately $10^{5}$ trainable parameters

### Appendix A.3. Summary of Evaluation Support Definitions

Table A3 provides a compact summary of the evaluation support sets defined in Section 3.8 and audited in Section 4.1.

**Table A3:** Evaluation support definitions.

Support Set	Definition	Main Use
$S_{\mathrm{grid}}$	All road segment–time positions in the test period	Reference test grid
$S_{\mathrm{target}}$	Positions with observed target speed, $m_{t,i}=1$	Primary relaxed evaluation
$S_{\mathrm{input}}$	Positions with a complete 12-step historical input window	Input completeness audit
$S_{\mathrm{strict}}$	$S_{target}\cap S_{input}$	Strict sensitivity audit
$S_{\mathrm{DP}}$	$S_{target}\cap S_{DailyProfile-valid}$	DailyProfile-common sensitivity check
Own-valid support	Method-specific valid prediction positions	Coverage-limited baseline reporting

## Appendix B

### Appendix B.1. Strict Evaluation Sensitivity Results

Section 4.8.1 presents the strict-versus-relaxed comparison using a scatter plot. Table A4 reports the corresponding numerical MAE values under the strict Evaluation-eligible support.

**Table A4:** Strict evaluation sensitivity results.

Model	Strict MAE (m/s)	Relaxed MAE (m/s)	Difference Δ
AGCRN	2.962	2.995	+0.033
DCRNN	3.029	3.055	+0.026
TT-LSTM C = 7	3.038	3.072	+0.034
TT-LSTM C = 4	3.040	3.077	+0.037
STGCN	3.158	3.177	+0.019
GWNet	3.468	3.494	+0.026

The positive differences indicate that relaxed evaluation is consistently harder than strict evaluation, while the broad ranking pattern is preserved.

### Appendix B.2. Multi-Seed Stability Check

To examine whether the small difference between TT-LSTM C = 4 and C = 7 is sensitive to random initialization, a multi-seed stability check was conducted for the TT-LSTM probe variants. The variants were trained with three random seeds and evaluated on the common strict support.

The mean difference between C = 4 and C = 7 is 0.0014 m/s under the strict support. Together with the small standard deviations reported in Table A5, this indicates that the C = 7 variant provides a stable but operationally small improvement over the baseline C = 4 configuration.

**Table A5:** Multi-seed stability for TT-LSTM feature ablation.

Variant	Mean Strict MAE (m/s)	Standard Deviation
TT-LSTM C = 4	3.0408	0.0008
TT-LSTM C = 7	3.0394	0.0016
